# Analysis of the performance of the CorneAI for iOS in the classification of corneal diseases and cataracts based on journal photographs

**DOI:** 10.1038/s41598-024-66296-3

**Published:** 2024-07-05

**Authors:** Yosuke Taki, Yuta Ueno, Masahiro Oda, Yoshiyuki Kitaguchi, Osama M. A. Ibrahim, Naohiko Aketa, Takefumi Yamaguchi

**Affiliations:** 1 Department of Ophthalmology, Tokyo Dental College Ichikawa General Hospital, 5-11-13, Sugano, Ichikawa, Chiba 272-8513 Japan; 2 Department of Ophthalmology, Faculty of Medicine, University of Tsukuba, Tsukuba, Ibaraki Japan; 3 Scholarly Information Division, Information Technology Center, Nagoya University, Nagoya, Aichi Japan; 4 Graduate School of Informatics, Nagoya University, Nagoya, Aichi Japan; 5 Department of Ophthalmology, Osaka University Gradual School of Medicine, Suita, Osaka Japan; 6 Clinical and Translational Research Center, Keio University Hospital, Shinjuku, Tokyo Japan

**Keywords:** CorneAI, Artificial intelligence, iPhone, Smartphone, Cornea diseases, Eye diseases, Eye manifestations

## Abstract

CorneAI for iOS is an artificial intelligence (AI) application to classify the condition of the cornea and cataract into nine categories: normal, infectious keratitis, non-infection keratitis, scar, tumor, deposit, acute primary angle closure, lens opacity, and bullous keratopathy. We evaluated its performance to classify multiple conditions of the cornea and cataract of various races in images published in the *Cornea* journal. The positive predictive value (PPV) of the top classification with the highest predictive score was 0.75, and the PPV for the top three classifications exceeded 0.80. For individual diseases, the highest PPVs were 0.91, 0.73, 0.42, 0.72, 0.77, and 0.55 for infectious keratitis, normal, non-infection keratitis, scar, tumor, and deposit, respectively. CorneAI for iOS achieved an area under the receiver operating characteristic curve of 0.78 (95% confidence interval [CI] 0.5–1.0) for normal, 0.76 (95% CI 0.67–0.85) for infectious keratitis, 0.81 (95% CI 0.64–0.97) for non-infection keratitis, 0.55 (95% CI 0.41–0.69) for scar, 0.62 (95% CI 0.27–0.97) for tumor, and 0.71 (95% CI 0.53–0.89) for deposit. CorneAI performed well in classifying various conditions of the cornea and cataract when used to diagnose journal images, including those with variable imaging conditions, ethnicities, and rare cases.

## Introduction

The cornea and crystalline lenses are crucial for focusing light onto the retina and maintaining optimal vision. Pathological conditions of the ocular media, such as, corneal opacity, infectious keratitis and cataracts, are the leading causes of vision impairment, affecting 75 million people worldwide (15 million with blindness (presenting visual acuity of < 3/60 in the better eye) and 60 million with moderate-to-severe vision impairment)^1,2^. Corneal diseases and cataracts are considered as avoidable vision loss with early detection and timely medical intervention^1–3^. However, real-world diagnosis and treatment depend on the availability of skilled ophthalmologists. Despite recent medical progress, the number of patients with avoidable blindness due to corneal diseases and cataracts continues to increase as the global population grows and ages, owing to the limited number of experienced ophthalmologists^3,4^.

In recent years, the integration of artificial Intelligence (AI) into healthcare has emerged as a transformative force, revolutionizing diagnostic processes and patient care^5,6^. AI applications are now used in various medical fields, including ophthalmology^7,8^.Advancements in AI have significantly benefited ophthalmology, enabling the use of fundus images to identify retinal diseases, such as retinal detachment^9^, age-related macular degeneration^10^, diabetic retinopathy^11,12^, and glaucoma^13,14^. For anterior segment diseases, studies have reported the classification of bacterial and fungal keratitis using anterior segment slit-lamp images^15,16^. Diagnosing corneal diseases requires a skilled ophthalmologist to examine the patient’s cornea using a slit-lamp microscope or slit-lamp imaging. Although these AI techniques have achieved promising results, their ability to differentiate between multiple corneal diseases remains limited^17^.

Furthermore, previous studies on AI applications in ophthalmology often rely on specialized medical equipment, such as slit-lamp microscopes, fundus cameras, and anterior segment optical coherence tomography (OCT) devices. Limited availability of such equipment in medically underserved areas leads to delays in diagnosis and treatment. AI that can be used for different races is necessary. One notable breakthrough is the integration of AI-powered diagnostic tools into everyday devices, such as smartphones^18,19^. Recently, we developed an AI-powered smartphone application called “CorneAI” that can categorize anterior eye images^20^. However, its performance in classifying diverse, real-world images captured under various conditions remains unclear. Ophthalmology journals, such as the *Cornea* journal (Lippincott, Philadelphia, PA, USA), contain anterior eye images for various diseases, races, and imaging conditions. This AI was created using a Japanese image dataset and we did not evaluate whether it can be utilized for a variety of races. Therefore, this study evaluated the performance of CorneAI in classifying anterior eye diseases published in the *Cornea* journal over 12 years from 2011 to 2022.

## Results

A total of 357 anterior segment images met the inclusion criteria for this study. The demographics characteristics of patients categorised by disease are summarised in Table 1. Infectious keratitis was the most prevalent category (122 eyes), followed by scar (73 eyes) and tumor (72 eyes). Notably, images related to APAC, lens opacity, and bullous keratopathy were not observed in the *Cornea* journal between 2011 and 2022 (Table 1).

**Table 1:** Total number of images across the nine disease categories.

	Normal	Infectious keratitis	Non-infection keratitis	Scar	Tumor	Deposit	APAC	Lens opacity	Bullous keratopathy
Total no	15	122	26	73	72	45	0	0	4

*APAC* Acute primary angle closure.

### Performance of CorneAI

The total PPV for the highest-ranking predictive score was 0.75. Its performance exceeded 0.92 when the third classification candidate was included. Infectious keratitis demonstrated the highest individual disease PPV at 0.91. PPVs for other categories were normal eye (0.73), non-infection keratitis (0.42), scar (0.72), tumor (0.77), and deposit (0.55) (Table 2). When the third classification candidate was included, the classification performance for each disease exceeded 0.80.

**Table 2:** Total number of images and performance of nine disease categories when included within the first, second, and third predictive scores.

	Top 1		Top 1–2		Top 1–3	
	Total no	PPV	Total no	PPV	Total no.	PPV
Normal	11	0.73	12	0.80	12	0.80
Infectious keratitis	111	0.91	119	0.97	119	0.97
Non-infection keratitis	11	0.42	21	0.80	22	0.84
Scar	53	0.72	65	0.89	67	0.91
Tumor	55	0.76	68	0.94	69	0.95
Deposit	25	0.55	33	0.55	38	0.84
Bullous keratopathy	2	0.5	3	0.75	3	0.75

*PPV* Positive predictive value.

**Figure 1:**
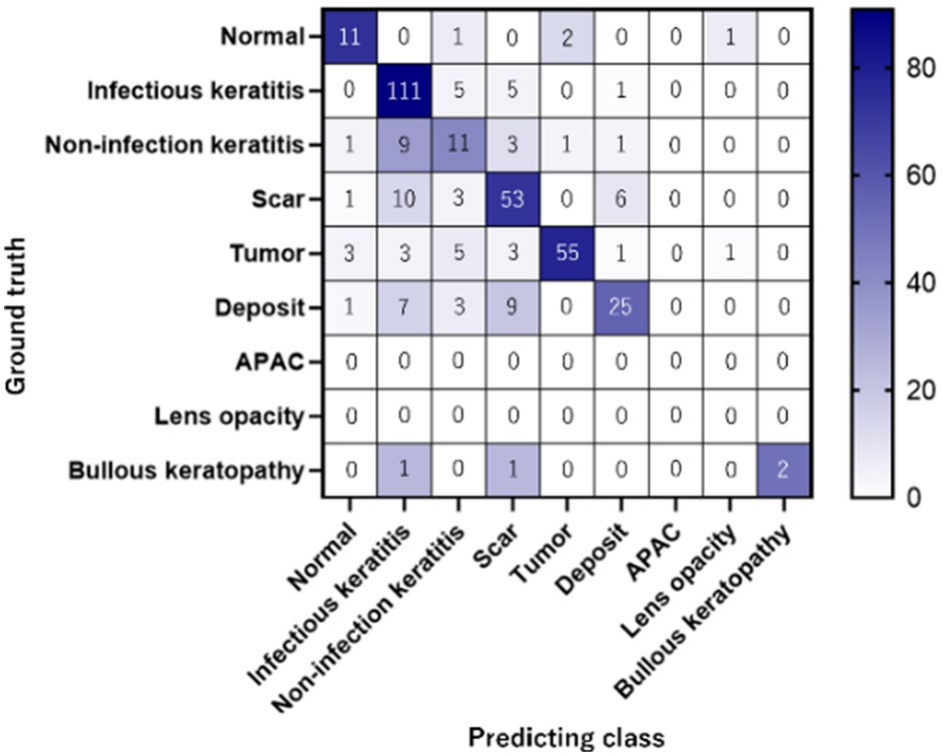
Confusion matrices describing nine corneal disease/cataract categories. This chart shows the confusion matrix for nine corneal disease/cataract classifications. Element (*i, j*) of each confusion matrix represents the empirical probability of the predicting class* j* given that the ground truth was class *i*.

Figure 1 shows the confusion matrices for the nine diseases. Elements (*i, j*) of each confusion matrix represent the empirical probability of the predicting class *j*, while the ground truth is class *i*. There were some cases wherein CorneAI for iOS misclassified infectious keratitis as non-infectious keratitis, scarring or deposition.

**Figure 2:**
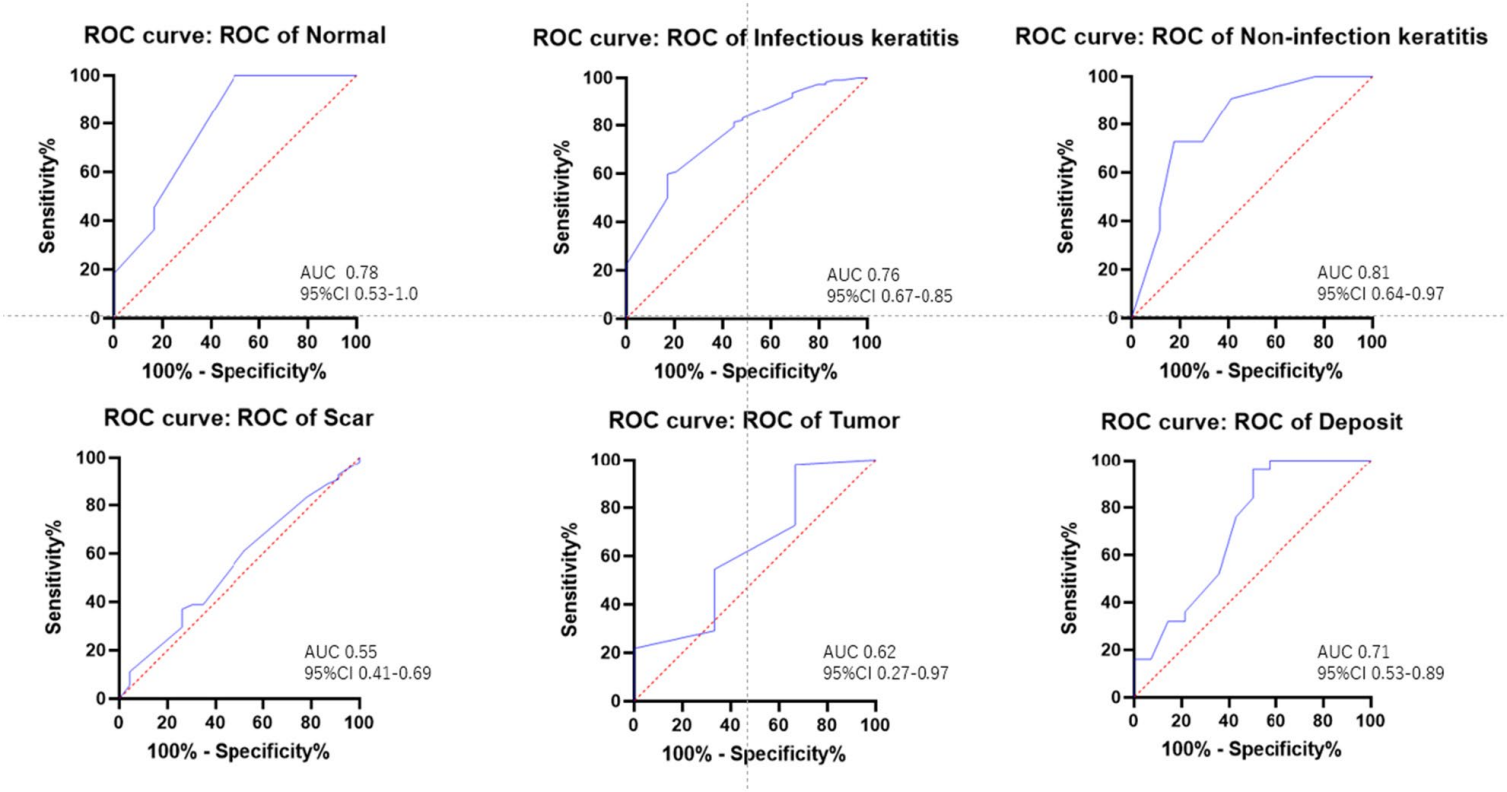
Performance of deep learning algorithm to classify nine corneal disease/cataract categories. The ROC curves indicate the performance of CorneAI for each category. ROC: receiver operating characteristic.

The AUC of the proposed deep learning algorithm were: 0.78 (95% CI 0.53–1.00) for normal eye, 0.76 (95% CI 0.67–0.85) for infectious keratitis, 0.81 (95% CI 0.64–0.97) for non-infection keratitis, 0.55 (95% CI 0.41–0.69) for scar, 0.62 (95% CI 0.27–0.97) for tumor, and 0.71 (95% CI 0.53–0.89) for deposit (Fig. 2). The sensitivity and specificity were 1.00 (95% CI 0.741–1.00) and 0.500 (95% CI 0.187–0.812) for normal eye, 0.607 (95% CI 0.514–0.692) and 0.793 (95% CI 0.616–0.901) for infectious keratitis, 0.727 (95% CI 0.434–0.902) and 0.823 (95% CI 0.589–0.938) for non-infection keratitis, 0.370 (95% CI 0.254–0.503) and 0.739 (95% CI 0.535–0.874) for scar, 0.981 (95% CI 0.903–0.999) and 0.333 (95% CI 0.171–0.881) for tumor, 1.00 (95% CI 0.866–1.00) and 0.428 (95% CI 0.212–0.674) for deposit, and 1.00 (95% CI 0.177–1.00) and 1.00 (95% CI 0.177–1.00) for bullous keratopathy, respectively (Table 3).

**Table 3:** Performance of CorneAI for disease categories.

	Sensitivity (95% CI)	Specificity (95% CI)
Normal	1.000 (0.741 to 1.000)	0.500 (0.187 to 0.812)
Infectious keratitis	0.607 (0.514 to 0.692)	0.793 (0.616 to 0.901)
Non-infection keratitis	0.727 (0.434 to 0.902)	0.823 (0.589 to 0.938)
Scar	0.370 (0.254 to 0.503)	0.739 (0.535 to 0.874)
Tumor	0.981 (0.903 to 0.999)	0.333 (0.171 to 0.881)
Deposit	1.000 (0.866 to 1.000)	0.428 (0.212 to 0.674)
Bullous keratopathy	1.000 (0.177 to 1.000)	1.000 (0.177 to 1.000)

*CI* Confidence interval.

The total PPV for the highest-ranking predictive score was 0.75 and 0.73 when classifying PNG images with the photographic mode and the journal images of *Ophthalmology* with the real-time mode, respectively (Supplementary Fig. 1). The PPV for APAC was 0.81 when classifying APAC images from Japanese textbooks.

### Classification errors

**Figure 3:**
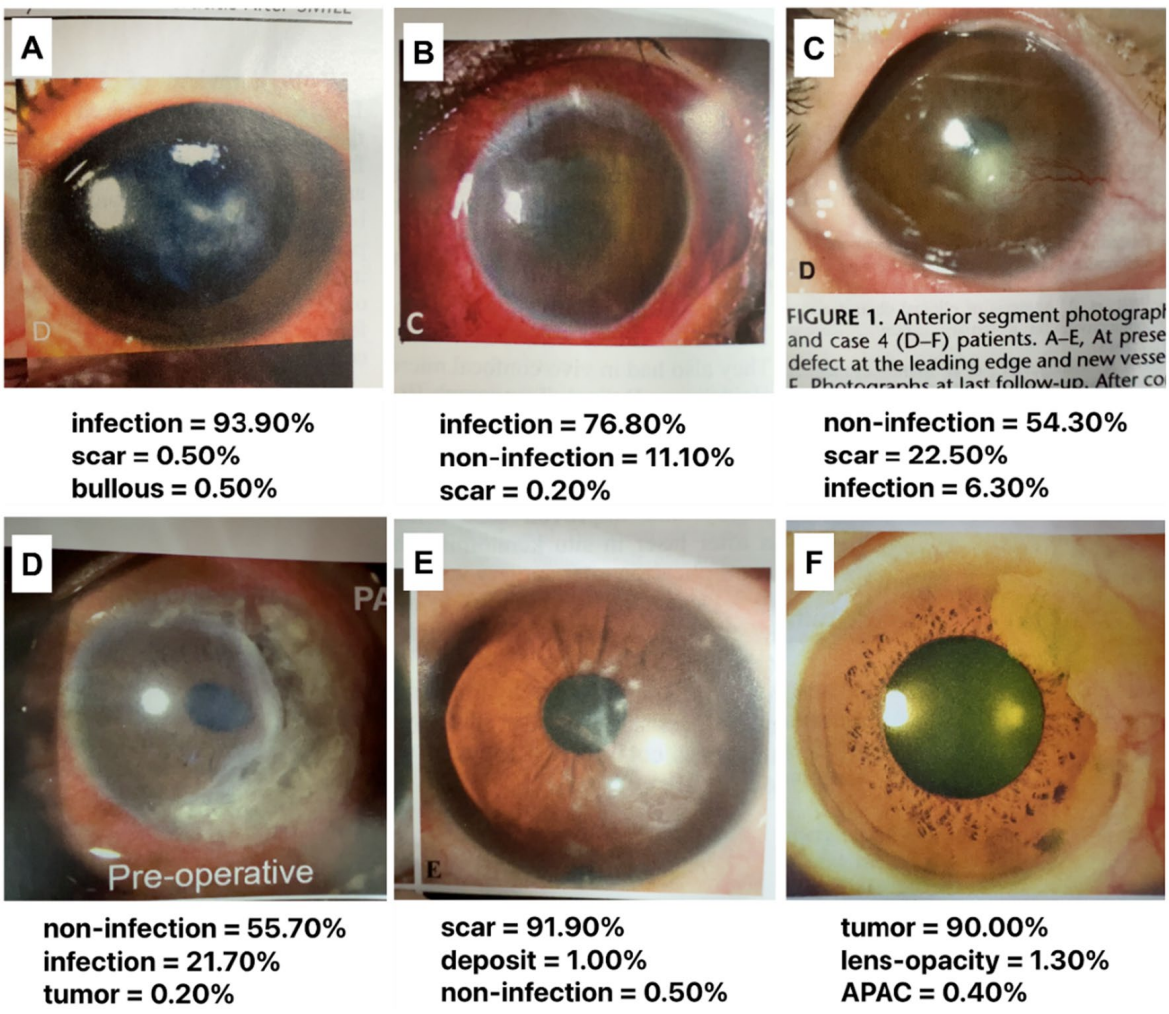
Representative examples of images correctly classified by CorneAI. (**A**) Image of mycobacterium keratitis classified as “infection.” (**B**) Image of bacterial keratitis classified as “infection.” (**C**) Image of phlyctenular keratitis classified as “non-infection.” (**D**) Image of Mooren ulcer classified as “non-infection.” (**E**) Image of corneal scar classified as “scar.”(**F**) Image of squamous cell carcinoma classified as "tumor".

Figure 3 shows representative images of correctly classified infectious keratitis (a, b), non-infection keratitis (c, d), scar (e), and tumor (f). CorneAI effectively categorised typical infectious keratitis cases and identified rarer instances, such as *Mycobacterium keratitis* and *Acanthamoeba keratitis*.

**Figure 4:**
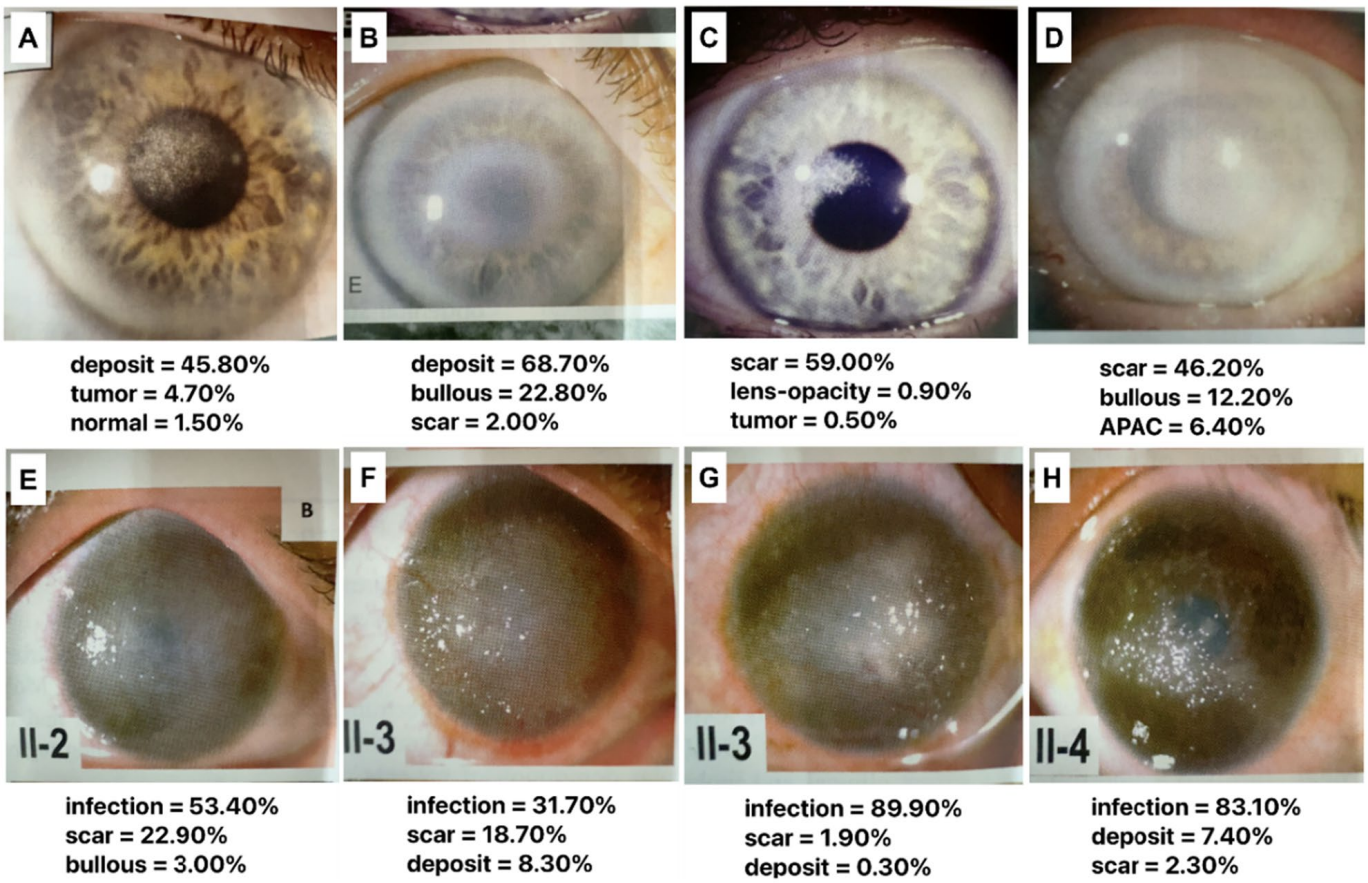
Representative examples of misclassified images by CorneAI. (**A**, **B**) Images of Schnyder corneal dystrophy correctly classified as “deposit.” (**C**, **D**) Images of Schnyder corneal dystrophy misclassified as “scar.” (**E**–**H**) Images of gelatinous drop-like dystrophy misclassified as “infection”.

Figure 4 illustrates examples of misclassified images. In Schnyder corneal dystrophy (a-d), some lesions were correctly classified as deposits, whereas others were misclassified as scars (50%). Similarly, most gelatinous drop-like corneal dystrophy (GDLD, e–h) were misclassified as infectious keratitis, potentially owing to the small number of GDLD examples in the CorneAI training dataset.

**Figure 5:**
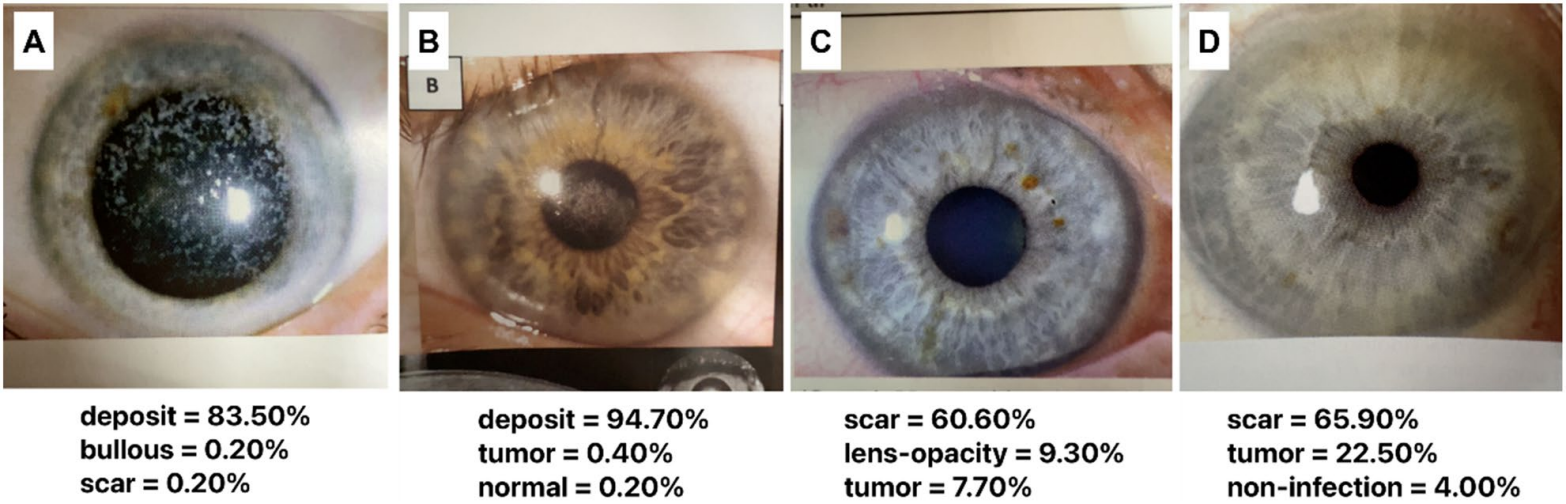
Classification of images with blue irises. (**A**) Image of granular corneal dystrophy with blue iris classified as “deposit.” (**B**) Image of Schnyder corneal dystrophy with blue iris classified as “deposit”. (**C**) Image of normal eye with blue iris misclassified as “scar.” *(**D**) Image of normal eye with blue iris misclassified as “scar.”

Since CorneAI was trained using images from brown-eyed Japanese, its performance in other races must be validated. We tested it on images published in the *Cornea* journal featuring Caucasian with blue, gray, and hazel eyes. CorneAI effectively identified abnormalities in blue-eyed individuals, accurately classifying cases like granular corneal dystrophy and Schnyder corneal dystrophy as deposits (Fig. 5A, B). However, normal eyes of blue-eyed individuals were prone to misclassify as scars or tumors (Fig. 5C, D).

## Discussion

We evaluated the classification performance of CorneAI using typical anterior segment images published in the *Cornea* journal, encompassing difference races and diseases. The total PPVs for the top 1 and 1–3 classifications were 0.75 and 0.92, respectively, comparing favourably with previously reported smartphone-based classifications^23^. CorneAI’s performance for infectious keratitis was particularly high (PPV = 0.91), while non-infection keratitis and deposit classifications were lower (0.42 and 0.55, respectively). This disparity may be attributed to race differences and limited training datasets for rare diseases. Classification performance was ensured under various conditions using the real-time mode, which is easy for everyone to use. Smartphone real-time-based classification holds the potential to revolutionize conditions of the cornea and cataract diagnosis.

Gu et al.^17^recently reported deep learning systems for detecting infectious keratitis, non-infection keratitis, corneal dystrophy or degeneration, and corneal neoplasms using slit-lamp images. The area under the ROC curve of the algorithm for each type of corneal disease was > 0.91. The PPV for the correct classification of infectious keratitis was 0.88. Li et al.^19^ reported an algorithm for classifying infectious keratitis, other corneal diseases, and normal eyes using a smartphone (all AUCs > 0.96). Here, the PPV of the correct classification for infectious keratitis was 0.94. Notably, the classification performance of our algorithm for infectious keratitis was comparable with that of previous studies. Infectious keratitis can be characterized by hyperemia, ulceration, and infiltration and these may be inherently easier for AI to identify. However, some images of late-stage infectious keratitis were classified as scarring, suggesting that images with minimal hyperemia, ulceration, and infiltration (which may represent healing stages) with scarring could be prone to misclassification.

Compared with Gu et al.^17^, our study recorded lower PPVs for non-infection keratitis (0.42 vs. 0.85), deposit (0.55 vs. 0.84) and tumor (0.77 vs. 0.89). One-third of the images of non-infection keratitis were incorrectly classified as infectious keratitis. However, we postulate that the low PPV in the current study was due to the rare non-infection keratitis cases published in *Cornea*, and not because of the incorrect classification of typical images from different races and imaging conditions. For example, CorneAI accurately diagnosed typical peripheral ulcerative keratitis, phlyctenular keratitis, and neurotrophic keratitis. In contrast, rare non-infection keratitis due to conditions such as Takayasu diseases^21^, Henoch-Schönlein purpura^21^, or Buerger disease^21^ were incorrectly classified as scar. Corneal melt following SARS-CoV-19 vaccination was classified as infectious keratitis and concluded to be non-infection keratitis, in which the authors suspected an infection with culture-negative results^22^. In actual clinical practice, corneal specialists tend to make mistakes in rare non-infection keratitis cases. Therefore, the performance of CorneAI should not be viewed as inferior to human diagnosis.

Approximately half of the deposit images were misclassified as infectious keratitis or scar (Fig. 4**)**. The crystalline-like round deposition of typical Schnyder corneal dystrophy was correctly classified as deposit (Fig. 4A, B). In contrast, in cases with Schnyder dystrophy, irregular asymmetric opacities (Fig. 4C) or blurred white opacities (Fig. 4D) were incorrectly classified as scar, which may be difficult for CorneAI to classify. Similarly, approximately one-third of the GDLD cases were incorrectly classified as infectious keratitis (Fig. 4E–H). Most of them were typical mulberry type, and AI may have incorrectly classified the glossy amyloid on the ocular surface as an infectious infiltrate^23^. These two categories reduced the classification performance. The poor classification performance of Schnyder corneal dystrophy and GDLD may be due to limited training data at the time of algorithm creation. It will be necessary to increase the number of cases and variations in disease findings to improve the algorithm in the future. This underscores the importance of recognizing specific diseases where AI may not excel, necessitating caution and clinical judgement when utilizing AI-based classification tools.

This study uses images from an international journal, including some with blue irises. Our AI was initially trained on anterior-segment images that primarily featured Japanese individuals with brown irises. CorneAI's performance varied when analyzing blue irises. While some eyes with normal blue iris were incorrectly classified as having scar (Fig. 5C, D), CorneAI accurately classified cases with deposits and blue irises (Fig. 5A, B). This suggests that AI can correctly classify conditions when noticeable lesions exist, regardless of the iris color. However, in normal eyes with blue irises, the distinct color of the iris itself seems to distract the AI, leading to false classifications. When we collected and classified images of 12 cases of normal eyes with blue, gray, and hazel irises, the PPV was only 0.16 (Supplementary Fig. 2). CorneAI needs to be retrained with a dataset encompassing blue iris images and then re-evaluate its performance to improve its accuracy.

Infectious keratitis is an emergency disease, and early diagnosis determines the prognosis owing to its rapid progression^24,25^. APAC is an emergency disease as it causes blindness if not treated early. Although there were no images in the *Cornea* journal, the classification of APAC images from textbooks was highly accurate, with a PPV of 0.81. As an automated screening tool, this system could be applied in developing countries or areas without access to medical resources to identify keratitis in its early stages and provide timely referrals for positive cases, potentially preventing corneal visual impairment. Furthermore, ocular surface tumors, such as conjunctival squamous cell carcinoma, may metastasize and cause death^26^. Non-infection keratitis also requires early treatment for a good visual prognosis. CorneAI, potentially available to all smartphone users in the future, aims to assist people or non-ophthalmology doctors unfamiliar with eye diseases. CorneAI not only adheres to necessary regulations but also places a high priority on user privacy, ensuring that personal health data is handled with the utmost confidentiality. We compared the classification performance in the real-time and photographic modes; the PPVs of both methods were similar (Supplementary Fig. 1). The real-time and photographic modes are available in CorneAI for iOS. The real-time mode would be useful for patients’friends or family members. In contrast, the photographic mode would be useful in remote AI-assisted diagnosis, sending anterior segment images captured using smartphones. It simplifies disease classification into urgent, needs further examination, or normal. Conditions such as infectious keratitis and acute glaucoma, classified as urgent, encourage prompt hospital visits, ensuring timely treatment^20^. However, using CorneAI on individual smartphones introduces potential limitations. Images captured with scratched camera’s lenses may induce artifacts affecting classification accuracy and make performance dependent on individual device capabilities. Therefore, testing CorneAI across various smartphone software versions and models is necessary.

We changed the PyTorch model to CoreML to run the “You Only Look Once” version 5 (YOLO V.5) on the Apple neural engine in iOS. This conversion affects the accuracy, but Jens et al. reports that CoreML greatly reduces the latency of a machine learning model and only performed around 1% worse on average^27^. Programmatic problems do not cause accuracy loss and, as aforementioned, rare cases and cases with blue irises were the causes of low accuracy.

Our study had several limitations that should be considered. First, while selecting images for classification, images with poor quality or decentered images of peripheral corneal area were excluded. This selection bias could potentially underestimate the AI’s performance in real-world settings. In clinical practice, acquiring clear images is essential. However, our study conducted classifications in the real-time mode, which may have mitigated issues related to variations in image acquisition conditions. Second, our current algorithm cannot display heatmaps. This hampers our ability to pinpoint the specific features or regions that influenced the AI’s incorrect classifications. We are actively working on introducing a heatmap visualization feature to address this limitation. This feature would highlight abnormal corneal regions in images, aiding the clinical review and verification of the AIs classifications.

In conclusion, we evaluated the classification performance of anterior segment images from the *Cornea* journal using CorneAI, a smartphone-based AI model installed in an iPhone13Pro, for categorising anterior segment diseases from diverse image sources. This app would be useful for classification and can be installed on portable devices, such as smartphones, which could be helpful in triaging conditions of the cornea and cataract in developing countries and areas with limited access to medical resources.

## Materials and methods

This study was approved by the Institution Review Board of the Japanese Ophthalmological Society (Protocol number: 15000133-20001). All procedures conformed to the tenets of the Declaration of Helsinki and the Japanese Guidelines for Life Science and Medical Research.

### CorneAI and image selection

We developed an AI model named CorneAI using the YOLO V.5 architecture to classify the condition of the cornea and cataract into nine categories: normal, infectious keratitis, non-infection keratitis, scar, tumor, deposit, acute primary angle closure (APAC), lens opacity, and bullous keratopathy^20^. We retrieved the anterior segment images from the international professional journal (*Cornea*) between 2011 (30[1]) and 2023 (42[12]). Exclusion criteria included: (1) images with slit light or fluorescein staining; (2) monochrome images; (3) images obtained after keratoplasty; (4) low-quality images that were decentered or had inadequate light exposure.

Anterior eye images were classified using real-time mode of CorneAI installed on an iPhone 13 Pro smartphone (Apple Inc. Cupertino, California, USA). Smartphone images were captured using the super macro mode of an iPhone 13 Pro under the following conditions: (1) under standard room illumination (300 LUX); (2) a distance of approximately 3–5 cm between the image and the iPhone cameras; and (3) clear focus on the image (Videos 1 and 2). Images of the paper journal were directly retrieved with a smartphone. In real-time mode, the top three classification candidates for the image were displayed. Images were captured at the same location in the hospital, and the top three disease candidates with the highest predictive scores were recorded. We extracted some images in the PNG format and classified them using the photographic mode of CorneAI. Furthermore, we retrieved images from the international professional journal *Ophthalmology* to confirm whether accuracy is guaranteed with other journals, we also classified APAC images from some textbooks.

### Predictive score calculation

During AI model testing, the estimated categories and corresponding predictive scores of the predicted bounding boxes were calculated. The category with the highest predictive score was selected for the final classification. The predictive scores were calculated using the sigmoid function in AI models. In the final layer (output layer) of deep learning (DL)-based AI models, the sigmoid function is applied to the feature value provided to the final layer, which is represented by:

$${s}_{b,c}\left({x}_{b,c}\right)=\frac{1}{1+{e}^{-{x}_{b,c}}},$$

where s_(b,c) is the predictive score, x_(b,c) is the feature value provided to the final layer, b is the index of the estimated bounding boxes, and c = 1,…,9 is the index of the categories.

### Data analysis

The classification performance of each disease was evaluated, and the positive predictive value (PPV), sensitivity, and specificity of each category were calculated. The PPV including all nine categories was defined as “total PPV”. We determined ROC curves for the predictive scores of the nine categories. We also calculated the areas under the curves (AUCs) and their 95% confidence intervals (CIs). Curves closer to the top left quadrant indicate a better performance level than those close to the baseline. Statistical analyses were performed using Prism (version 6.04) for Windows software (Graphpad Software, Inc., San Diego, CA, USA).

### Inform consent

The review committee stated that patient consent was not required for the retrospective study of slit-lamp microscopy images, because all slit-lamp images used in the study were published previously and deidentified.

### Ethical approval

This study was approved by the Institution Review Board of Japanese Ophthalmological Society (Protocol number: 15000133-20001). All the procedures conformed to the tenets of the Declaration of Helsinki and the Japanese Guidelines for Life Science and Medical Research.

### Meeting presentation

 We presented this study in 129th Japanese Society of Clinical Ophthalmology.

### Supplementary Information

Supplementary Legends.

Supplementary Figures.

Supplementary Video 1.

Supplementary Video 2.

## Data Availability

The data that support the findings of this study are available on request from the corresponding author (T.Y.). The data are not publicly available due to them containing information that could compromise research participant privacy/consent.

## References

[1] Flaxman SR (2017). Global causes of blindness and distance vision impairment 1990–2020: A systematic review and meta-analysis. Lancet Glob. Heal..

[2] Pascolini D, Mariotti SP (2012). Global estimates of visual impairment: 2010. Br. J. Ophthalmol..

[3] Bourne RRA (2021). Trends in prevalence of blindness and distance and near vision impairment over 30 years: An analysis for the Global Burden of Disease Study. Lancet Glob. Heal..

[4] Gupta N, Tandon R, Gupta S, Sreenivas V, Vashist P (2013). Burden of corneal blindness in India. Indian J. Commun. Med..

[5] Litjens G (2017). A survey on deep learning in medical image analysis. Med. Image Anal..

[6] Matheny ME, Whicher D, Thadaney Israni S (2020). Artificial intelligence in health care: A report from the National Academy of Medicine. JAMA J. Am. Med. Assoc..

[7] Rashidi P, Bihorac A (2020). Artificial intelligence approaches to improve kidney care. Nat Rev Nephrol..

[8] Chilamkurthy S (2018). Deep learning algorithms for detection of critical findings in head CT scans: A retrospective study. Lancet.

[9] Li Z (2020). Deep learning for detecting retinal detachment and discerning macular status using ultra-widefield fundus images. Commun. Biol..

[10] Burlina PM (2017). Automated grading of age-related macular degeneration from color fundus images using deep convolutional neural networks. JAMA Ophthalmol..

[11] Gulshan V (2016). Development and validation of a deep learning algorithm for detection of diabetic retinopathy in retinal fundus photographs. JAMA J. Am. Med. Assoc..

[12] Gargeya R, Leng T (2017). Automated identification of diabetic retinopathy using deep learning. Ophthalmology.

[13] Li Z (2018). Efficacy of a deep learning system for detecting glaucomatous optic neuropathy based on color fundus photographs. Ophthalmology.

[14] Li Z (2021). Deep learning for automated glaucomatous optic neuropathy detection from ultra-widefield fundus images. Br. J. Ophthalmol..

[15] Ghosh AK, Thammasudjarit R, Jongkhajornpong P, Attia J, Thakkinstian A (2022). Deep learning for discrimination between fungal keratitis and bacterial keratitis: DeepKeratitis. Cornea.

[16] Hung N (2021). Using slit-lamp images for deep learning-based identification of bacterial and fungal keratitis: Model development and validation with different convolutional neural networks. Diagnostics.

[17] Gu H (2020). Deep learning for identifying corneal diseases from ocular surface slit-lamp photographs. Sci. Rep..

[18] Freeman, K. *et al.* Algorithm based smartphone apps to assess risk of skin cancer in adults: systematic review of diagnostic accuracy studies. *BMJ* **368**,127 (2020). 10.1136/bmj.m127 PMC7190019 32041693

[19] Li Z (2021). Preventing corneal blindness caused by keratitis using artificial intelligence. Nat. Commun..

[20] Ueno, Y., Oda, M., Yamaguchi, T., Fukuoka, H., Nejima, R., Kitaguchi, Y., Miyake, M., Akiyama, M., Miyata, K., Kashiwagi, K., Naoyuki Maeda, J. S. & Hisashi Noma Kensaku Mori, T. O. Deep learning model for extensive smartphone-based diagnosis and triage of cataracts and multiple corneal diseases. *Br. J. Ophthalmol. Epub ahead of print.*10.1136/bjo-2023-324488 (2024) . 10.1136/bjo-2023-324488 PMC11503034 38242700

[21] Levitt AE (2015). Ocular inflammation in the setting of concomitant systemic autoimmune conditions in an older male population. Cornea.

[22] Khan TA (2021). Bilateral immune-mediated keratolysis after immunization with SARS-CoV-2 recombinant viral vector vaccine. Cornea.

[23] Ide T (2004). A spectrum of clinical manifestations of gelatinous drop-like corneal dystrophy in Japan. Am. J. Ophthalmol..

[24] Watson S, Cabrera-Aguas M, Khoo P (2018). Common eye infections. Aust. Prescr..

[25] Sharma A, Taniguchi J (2017). Review: Emerging strategies for antimicrobial drug delivery to the ocular surface: Implications for infectious keratitis. Ocul. Surf..

[26] Santoni A (2019). Management of invasive squamous cell carcinomas of the conjunctiva. Am. J. Ophthalmol..

[27] Ahremark, J. *et**al*. Benchmarking a machine learning model in the transformation from PyTorch to CoreML. *LiU Electronic Press* 33 (2022)

