# Supplementary material for: Analysis of the performance of the CorneAI for iOS in the classification of corneal diseases and cataracts based on journal photographs
Source: Sci Rep. 2024 Jul 5;14:15517. doi: 10.1038/s41598-024-66296-3 (PMC11226423; doi:10.1038/s41598-024-66296-3)
Supplement: Supplementary file 1 — Supplementary Legends. [file 41598_2024_66296_MOESM1_ESM.docx]

**Supplementary video legends**

**Video 1. Classification of infectious keratitis with CorneAI using the real-time mode.**

Anterior eye images appear to be captured in the center of the screen. Based on these images, CorneAI can determine the three most probable classifications. Infectious keratitis was correctly classified as the most probable option.

**Video 2. Classification of tumor with CorneAI using the real-time mode.**

Anterior eye images appear to be captured in the center of the screen. Based on these images, CorneAI can determine the three most probable classifications. Tumor was correctly classified as the most probable option.

**Supplementary figure legends**

**Figure 1. Comparison of the total PPV for the highest-ranking predictive score in three ways.**

The green bar graph represents PPV for the highest-ranking predictive score of the *Cornea* journal images using the real-time mode. The blue bar graph represents PPV for the highest-ranking predictive score of the *Cornea* journal images with the photographic mode. The red bar graph represents PPV for the highest-ranking predictive score of the *Ophthalmology* journal images with the real-time mode.

**Figure 2. Classification of normal images with blue, gray, and hazel irises.**

(a, b) Images of normal eyes classified as “normal.” (c) Images of normal eyes classified as “lens-opacity.” (d–f) Images of normal eyes classified as “scar.” (g, h) Images of normal eyes classified as “tumor.”
