# Supplementary figures and images for: Analysis of the performance of the CorneAI for iOS in the classification of corneal diseases and cataracts based on journal photographs
Source: Sci Rep. 2024 Jul 5;14:15517. doi: 10.1038/s41598-024-66296-3 (PMC11226423; doi:10.1038/s41598-024-66296-3)

**Supplementary Figure 1**


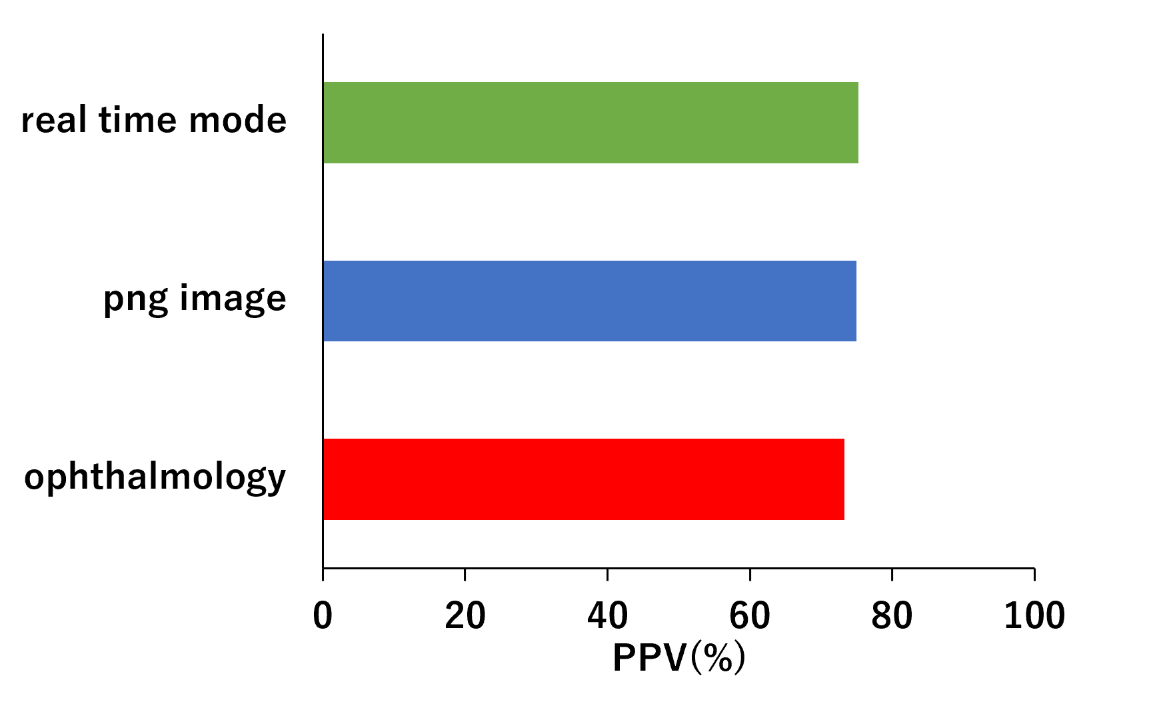


**Supplementary Figure 2**


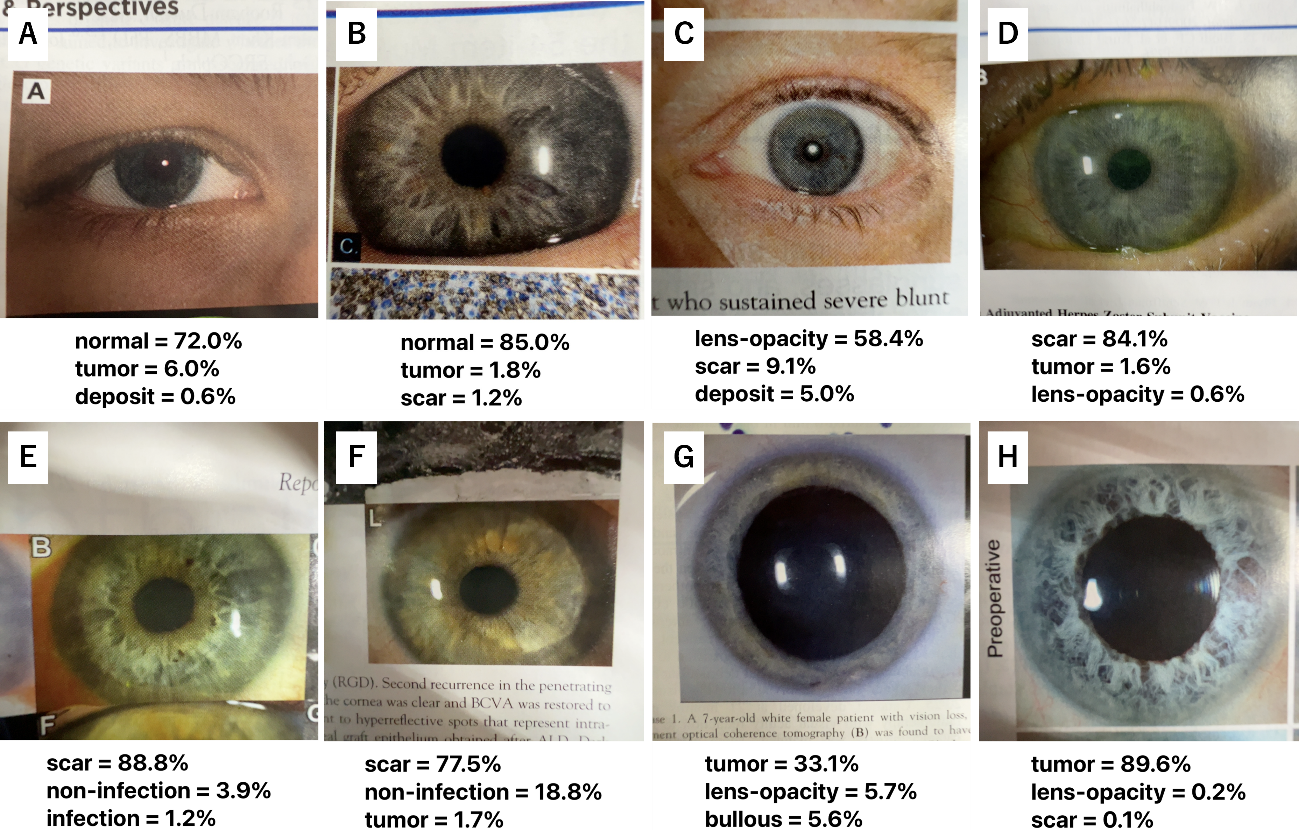

Supplement: Supplementary file 2 — Supplementary Figures. [file 41598_2024_66296_MOESM2_ESM.docx]
